# Bilateral cleft lip nasal deformity

**DOI:** 10.4103/0970-0358.59289

**Published:** 2009

**Authors:** Arun Kumar Singh, R. Nandini

**Affiliations:** Department of Plastic Surgery, C.S.M.M.U (upgraded K.G.M.C), Lucknow, India

**Keywords:** Bilateral cleft, primary nasal repair, cleft lip nose

## Abstract

Bilateral cleft lip nose deformity is a multi-factorial and complex deformity which tends to aggravate with growth of the child, if not attended surgically. The goals of primary bilateral cleft lip nose surgery are, closure of the nasal floor and sill, lengthening of the columella, repositioning of the alar base, achieving nasal tip projection, repositioning of the lower lateral cartilages, and reorienting the nares from horizontal to oblique position. The multiplicity of procedures in the literature for correction of this deformity alludes to the fact that no single procedure is entirely effective. The timing for surgical intervention and its extent varies considerably. Early surgery on cartilage may adversely affect growth and development; at the same time, allowing the cartilage to grow in an abnormal position and contributing to aggravation of deformity.

Some surgeons advocate correction of deformity at an early age. However, others like the cartilages to grow and mature before going in for surgery. With peer pressure also becoming an important consideration during the teens, the current trend is towards early intervention.

There is no unanimity in the extent of nasal dissection to be done at the time of primary lip repair. While many perform limited nasal dissection for the fear of growth retardation, others opt for full cartilage correction at the time of primary surgery itself. The value of naso-alveolar moulding (NAM) too is not universally accepted and has now more opponents than proponents. Also most centres in the developing world have neither the personnel nor the facilities for the same. The secondary cleft nasal deformity is variable and is affected by the extent of the original abnormality, any prior surgeries performed and alteration due to nasal growth.

This article reviews the currently popular methods for correction of nasal deformity associated with bilateral cleft lip, it's management both at the time of cleft lip repair and also secondarily, at a later date. It also discusses the practices followed at our centre.

## INTRODUCTION

The bilateral cleft lip nasal deformity may be primary, or secondary following variable growth of the nasal architecture after closure of the lip element. The severity of the primary nasal deformity is generally proportional to the severity of the cleft. The more extensive the cleft and the greater the protrusion of the premaxilla, the worse is the nasal deformity. An asymmetric cleft, as well as an asymmetric nasal deformity often pose a greater problem for nasal reconstruction.[1,2] Primary nasal deformity correction ensures balanced growth, better development of the nasal structures, and creates better conditions for a definitive cleft rhinoplasty. Secondary nasal deformity is a spectrum of deformities varying with the extent of the primary deformity, the surgery performed, and growth of the nasal architecture.[3] There are numerous ways to attempt correcting this deformity. We discuss the currently acceptable techniques along with our own experience; after recapitulating salient features of nasal deformity in bilateral clefts.

## ANATOMY

**Figure 1:**
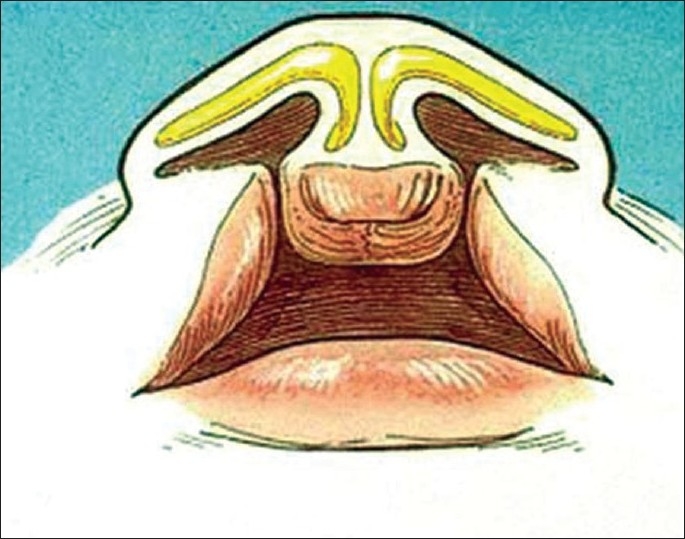
Findings in bilateral cleft lip nasal deformity

The features of the bilateral cleft lip nasal deformity primarily involve the columella, nostrils, the nasal tip and the lower lateral cartilages. [Figure 1]

- The columella is short. In severe cases it appears as though prolabium is attached to the nasal tip.
- The nasal tip is flat and broad and appears bifid.
- The nasal alae are flat and at times drawn in, in an S-shaped fashion.
- The bases of the alae are displaced laterally, and sometimes inferiorly and posterior.
- Both nostrils are horizontally oriented.
- The lower lateral cartilages are severely deformed.
- The medial crura are short and widely separated at the nasal tip.
- The lateral crura are flat and elongated.
- The dome is angled obtusely.
- The nasal floor is absent.

The columella, caudal end of the septum, and the anterior nasal spine are displaced inferiorly relative to the level of the alar bases.

The nasal tip and nostrils may be asymmetric.

**Figure 2:**
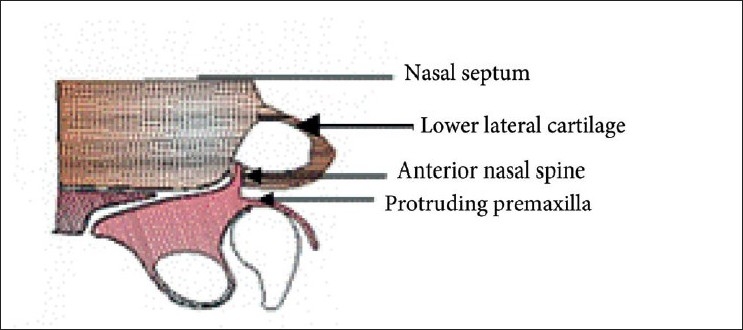
Arrangement of cartilage in newborn with bilateral cleft lip nasal deformity

An anatomical observation by Ralph A Latham[4] has brought out a different viewpoint regarding the arrangement of cartilage and soft tissues in the newborn with bilateral cleft lip and palate. They noticed that the medial crura of the alar cartilages look relatively normal. In lateral view, they are located anterior to the cartilaginous nasal septum and represent the normal skeletal component of the columella. However, they are obscured by the protrusive alveolar process of the premaxilla [Figure 2] The anterior nasal spine nestles between the flared out ends of the medial crura.

## REPAIR OF THE PRIMARY DEFORMITY

The goals of primary bilateral cleft lip nose surgery are:

- Closure of the nasal floor and sill
- Lengthening of the columella
- Repositioning of the alar base
- Achieving nasal tip projection
- Repositioning of the lower lateral cartilages
- Reorienting the nares from horizontal to oblique position

### Timing for correction

There are differences of opinion regarding the optimal timing for nasal repair. All surgeons perform limited nasal repair like nasal floor reconstruction and narrowing of alar base at the time of primary lip repair. The timing of final revision of soft tissue nasal deformity differs. Some surgeons take the child up for surgery at one year of age and others at four to seven years of age.

Limited correction of the nasal deformity done at the time of primary lip repair improves results of second stage of primary repair. At our centre, Bardach's method[5,6] is commonly used. We perform limited nasal correction, that is, reconstruction of nasal floor and sill at the time of lip repair. We correct the lower lateral cartilages before the child goes to school. Some centres prefer Salyer's two-stage technique.[5,6] The first stage is performed at three months of age. It consists of lip repair along with banking of tissue into the nasal floor. Nasal soft tissue correction is done at one year of age. It is considered to be the second stage of primary bilateral cleft lip-nose repair. Early correction of the nasal deformity would assure a more normal relationship of the columella-lip angle, better projection and definition of the tip, and also allow subsequent growth in a more normal anatomic relationship.

Mulliken[7,8] believes that the columella is not short but lies within the nose. He advocates primary repair of the nasal cartilage along with the lip repair at three to five months of age. We have currently adopted this principle. We perform total primary nasal repair at the time of lip repair. We have also incorporated presurgical nasoalveolar moulding (NAM) in our protocol to aid in single stage repair. This is because the size of the premaxillary segment and the extent of its protrusion vary considerably. In complete clefts, it is often necessary to retroposition the premaxilla before definitive lip repair.

The principle objective of presurgical NAM is to reduce the severity of the initial cleft deformity enabling the surgeon to enjoy the benefits associated with repair of an infant presenting with a minimal cleft deformity. The goals of NAM include lip segments that are almost in contact at rest, symmetrical lower lateral alar cartilages, and adequate nasal mucosal lining, which permits postsurgical retention of the projected nasal tip. Presurgical NAM also includes the nonsurgical elongation of the columella, centering of the premaxilla along the midsagittal plane, and retraction of the premaxilla in a slow and gentle manner to achieve continuity with the posterior alveolar cleft segments. Presurgical nasoalveolar moulding uses both an intraoral alveolar moulding device and nasal moulding prongs. Successful use of any presurgical orthopaedic devices requires a team approach.

If properly performed, presurgical nasoalveolar moulding can provide soft tissue expansion and mould the nasal architecture, thereby decreasing nasal deformity. Our initial results incorporating NAM with primary lip and nose repair have been encouraging.

### Bardach's technique: Steps for correction

**Figure 3:**
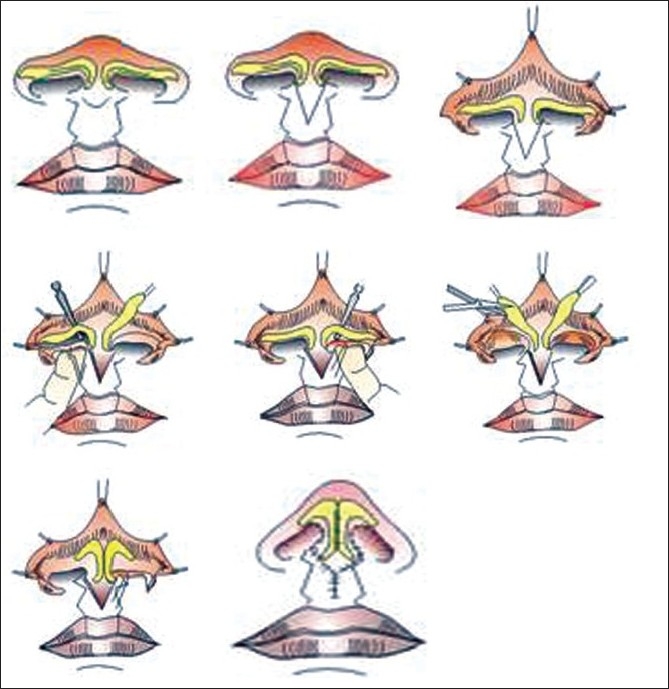
Bilateral cleft lip nose repair- Bardach's technique

Bardach's technique[5,6] [Figure 3] is performed at six to eight years of age. At the time of lip repair, construction of the floor of the nose and sill is done with symmetric repositioning of alar base. The second stage of primary repair involves lengthening of the columella and modification of the lower lateral cartilages by complete dissection of the lateral crura from the skin and nasal mucosa. Symmetric restructuring of both lateral crurae is done by trimming and shortening them. The lateral crura are sutured at the appropriate height to create a symmetric, projected support for the nasal tip.

**Figure 4:**
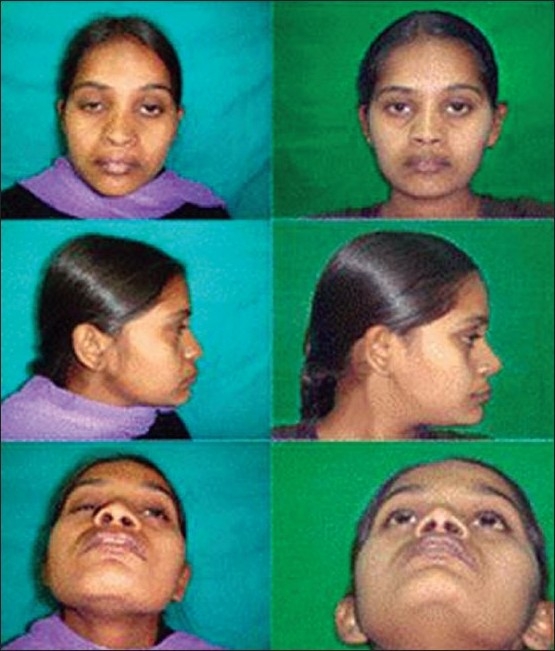
Correction of bilateral cleft lip nose deformity and lip revision using Bardach's principles

The principle of repair is to lengthen the columella and correct cartilage deformity at the same time. The lower lateral cartilage is freed from both skin and nasal mucosa and restructured so that the short medial crurae are lengthened and the lateral crura shortened, according to the desired shape of the nostrils. The lower lateral cartilages are sutured at the appropriate height to create a natural support for the projected nasal tip and lengthened columella. [Figure 4]

### Salyer's technique: Steps for correction [Figure 5]

**Figure 5:**
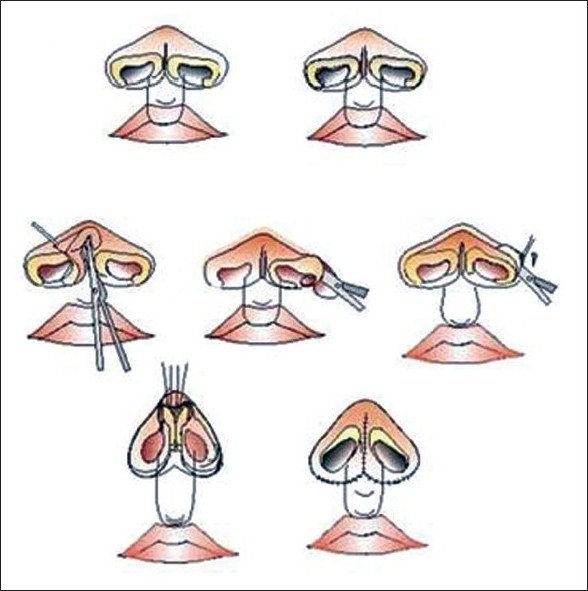
Bilateral cleft lip nose repair- Salyer's technique

**Figure 6:**
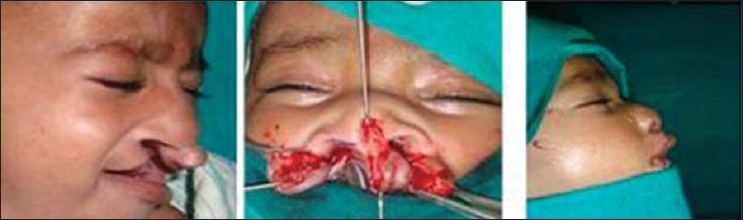
Cheiloplasty and primary nasal correction using Salyer's technique. Note the elongation of columella in the postoperative period

In this technique,[5,6] besides correction of the nasal floor and sill and symmetrical positioning of the alar bases at the time of lip repair, skin is banked in the floor of the nose for reconstruction of the columella at the time of nasal repair. This is undertaken at one year of age. [Figure 6] At this time, the columella is lengthened using banked skin from the nasal floor. The lateral crura of the lower lateral cartilages are dissected from the skin and nasal lining. The lining remains attached to the lower lateral cartilages in the alar dome region. The lower lateral cartilages are then realigned by lengthening the medial crura while shortening the lateral crura. The lining is transected between the septum and the columella to facilitate upward rotation of the medial crura and the columellar skin. Transection of the skin and mucosa on the nasal floor is performed to allow medial and superior rotation and lengthening of the columella. The lateral crura are sutured together under direct vision and at the appropriate height to establish cartilaginous support for a projected and symmetric nasal tip.

### One stage lip-nasal repair (Mulliken's method): Steps for correction

**Figure 7:**
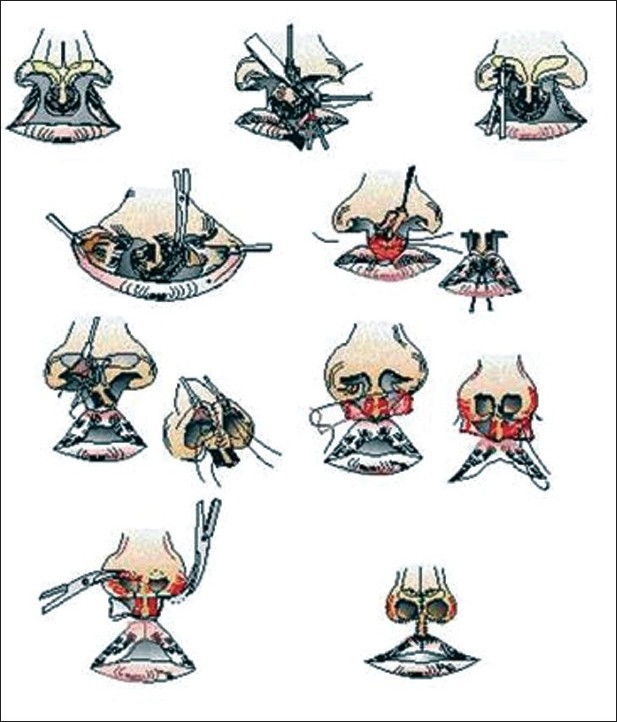
Mulliken method of single stage nasolabial repair

Mulliken[7,8] advocates synchronous repair of bilateral cleft lip nasal deformity during correction of lip. Primary nose correction at the time of lip repair is usually done at three to five months. [Figure 7]

The dislocated alar cartilages are visualized through bilateral rim incisions. A midline nasal tip incision may be given to facilitate the dissection. An interdomal mattress suture is placed to appose the middle crura. One or two mattress sutures suspend each lateral crus to the ipsilateral upper lateral cartilage. A cinch suture is placed through each alar base and is tightened until the interalar distance is less than 25 mm. Once the alar cartilages are in proper position, extra skin in the soft triangles should be excised. This resection narrows the tip, defines the columellar-lobular junction, elongates the nostrils, and narrows the columellar waist. There is also redundancy in the vestibular lining that becomes apparent after positioning the alar cartilages. Lenticular excision of this extra mucosa helps to obliterate the lateral vestibular web and supports the lateral crura.

**Figure 8:**
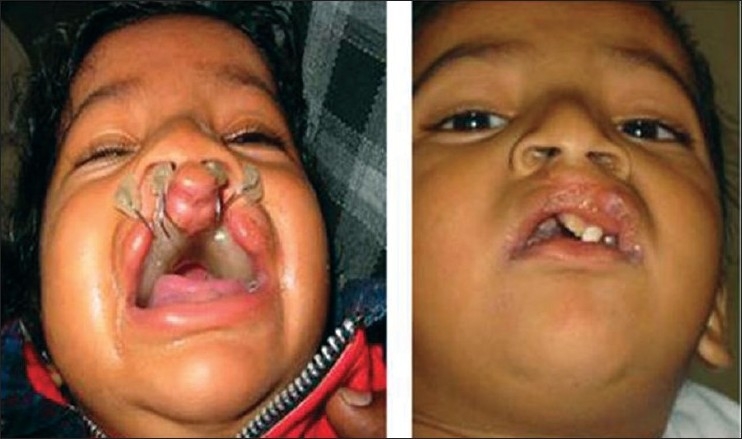
Bilateral cheiloplasty and no nasal dissection using preoperative NAM

**Figure 9:**
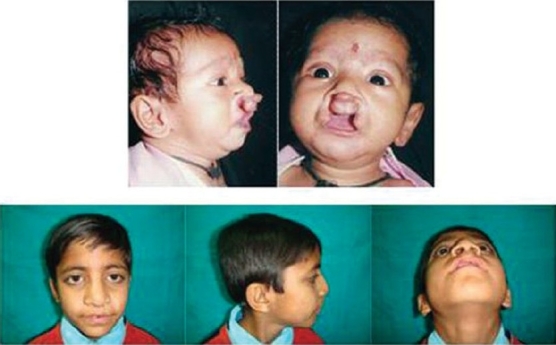
Single stage lip and nasal repair done at three months of age. Same patient at six years of age. Has correction done by semiopen technique)

In 1990, McComb[9,10] introduced the first primary repair in which the fundamental principle was bringing the domes of the lower lateral cartilages of the nose together into a normal anatomic relationship. This is based on the principle that the skeletal component of the columella is relatively normal in the cleft condition, but the covering skin is developmentally deficient. The columellar cartilages are covered and obscured by the alveolar process of the premaxillary segment. Hence, gradual retraction of skin will reveal the medial crura of the alar cartilages and establish a normal fleshy external termination of the septum of the nose. Primary columellar lengthening and nasal tip projection can be obtained solely by positioning the alar domes and sculpting the nasal soft tissues. Established reconstructive principles teach us to attempt to put structures back in their proper position. This has shifted focus to preoperative moulding of the alveolar plate to re-expose the covered columella and to perform primary nasal repair with lip repair [Figure 8] We have adopted this technique and are satisfied with our results [Figure 9]

### Evolution of single stage repair

In 1991, Trott and Mohan[11] began working in rural Malaysia where single-stage nasolabial repair was a necessity. They opened the nasal tip by lifting the prolabium and columella, dissected the anterior surface of the middle crura, and corrected the displaced alar cartilages, secured the alar cartilages in proper position and redraped them.

**Figure 10:**
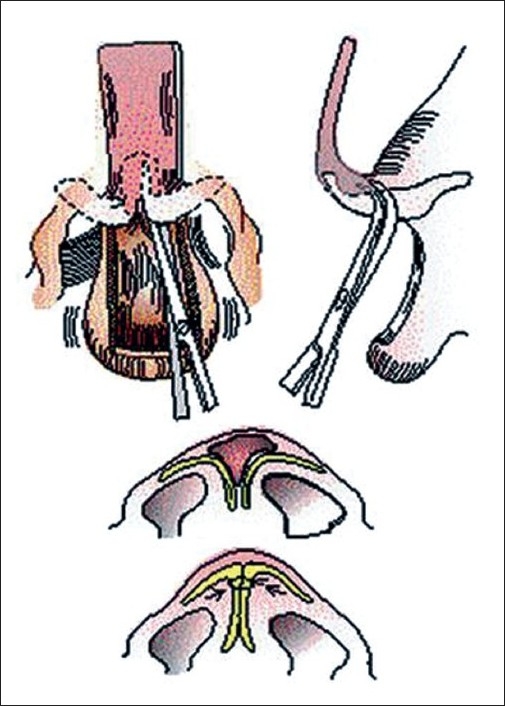
Retrograde dissection of nasal tip cartilages

The Japanese introduced presurgical moulding of the bilateral cleft nasal deformity. Cutting and co-workers[12,13] extended this scheme to preoperative stretching of the columella by an acrylic, double outrigger and prolabial band, attached to a palatal moulding plate, and secured to the cheeks with tape. They also use an open-tip approach. However they elevate the prolabial-columellar flap at a deeper plane (membranous septum) so that placement of interdomal sutures is visualized from the underside of the cartilages.[Figure 10]

### Combined Cutting and Mulliken method

Cutting's retrograde dissection of nasal tip cartilage works well in patients who have had successful presurgical moulding. In bilateral cleft lip patients who have received inadequate or no presurgical moulding of the nose and premaxilla, a retrograde approach needs to be combined with Mulliken's bilateral rim incision or bilateral Tajima's inverted-U incisions to obtain better exposure of alar cartilages for realignment.[14,15] The procedure begins using the retrograde approach to elevate the medial crura, columella, and prolabium as a composite flap. Both alar cartilages and their attached lining mucosa are dissected away from the overlying dome skin and fat using a retrograde approach.

To facilitate alar cartilage manipulation and lengthen the columella, bilateral marginal rim incisions are extended laterally inside the nostril rim. With these incisions it is possible to fully expose the alar cartilage and place interdomal mattress sutures to approximate the domes and medial crura. Another internal suture suspends each alar cartilage to the ipsilateral upper lateral cartilage. The skin at the marginal area is folded under to make the inner surface of the soft triangle, as described by Tajima in his “inverted-U” procedure.[16]

## REPAIR OF SECONDARY NASAL DEFORMITY

The secondary cleft nasal deformity is variable and is affected by the extent of the original abnormality, any prior surgeries performed and alteration due to nasal growth. Despite early intervention in the bilateral cleft at the time of the lip repair, lack of tip projection, an associated short columella, and displaced lower lateral cartilages often result.[17,18] However the extent of secondary deformity is definitely less in those who have undergone primary correction.

Our protocol for management of these patients is in two stages:

Correction during six to eight years of age

Definitive rhinoplasty when total nasal growth has occurred

### Management at six to eight years

The correction of nasal deformity at this age is limited to soft tissue repair and involves readjustment of lower lateral cartilage and correction of tip projection. Most children present with broad nasal tip and short nasal projection, though the spectrum of anomaly varies with previous intervention and extent of primary deformity. This is when we perform tip correction of cartilage framework using the modified open technique to provide access. Tip definition is adjusted using domal sutures supported by the columellar strut graft. The lower lateral cartilages are mobilized free from vestibular lining and outer skin and adjusted with interdomal and/or intradomal sutures. The lower lateral cartilage dome is elevated and hence the columella elongates and maintains that position in the long term. When required, a rigid cartilage strut graft is secured with sutures between the medial crura of the lower lateral cartilages. Initially, the projection will be prominent which later settles down and may even be slightly inadequate as adult size is attained. Additional tip support can be provided by placing septal grafts on the caudal side of the intermediate crura of the lower lateral cartilage during definitive rhinoplasty.

### Surgical management at 12-16 years

In all of our cleft patients, the final adjustments in nasal reconstruction are done at around 14-16 years in boys and in girls at 12-14 years or later whenever the patient presents. In patients requiring orthognathic surgery, rhinoplasty is done after maxillary advancement. The final rhinoplasty is done through an open approach. The lower lateral cartilages, which are often surrounded by scar, are carefully visualized. Septal abnormalities are corrected.[17] After any required adjustments in the nasal dorsum, septal cartilage can be obtained for use during reconstruction of the nose. The nasal labial angle is decreased by using a caudal tip graft. Symmetrical lower lateral cartilages are created by using a combination of intradomal and interdomal sutures and spanning sutures. Direct adjustment of the caudal border of the lower lateral cartilages is occasionally done. Osteotomy of the nasal pyramid is done to correct the nose defect if the deviation begins at the nasion, the nasal pyramid is wide, or the nasal dorsum is excessively prominent and requires reduction.
